# Was It Hydrophobia?

**Published:** 1894-04

**Authors:** 

**Affiliations:** Richland, Texas; Richland, Texas


For Texas Medical Journal.

### WAS IT HYDROPHOBIA?

BY DRS. BROWN AND BAILY, RICHLAND, TEXAS.

J. L. S.; age 37 years; male, of medium size, and very stout; healthy habits; regular occupation, cow-boy and farmer; of considerable industry and will-power; not excitable or easily influenced, was bitten by a pup (supposed to have distemper) on December 26, also two of his own and several school children; ages running from 7 to 10 years, respectively, were bitten. He pursued his usual occupation, plowing and driving cattle, until Monday, February 26th, which day he plowed hard on very rough ground, and, as he supposed, got too hot.

Tuesday he did not feel like work; complained of pain in right arm, elbow, and shoulder of same hand that was bitten, on second phalanx of the middle digit.

Tuesday evening he came to my office. I diagnosed rheumatism with malaria, and also noticed a great change in conversation, he appearing, at this time, very serious, while usually he was very jovial. I prescribed the usual remedies, pot. iodide and colchicum.

Wednesday, March 1st, 7 a. m., I was sent for hurriedly; messenger saying Mr. S—— has hydrophobia. Supposing it to be something unusual, I asked Dr. Bailey to accompany me. We found him sitting by the fire; had been smoking, but refused to eat and drink; and we were informed that he had sent for a *mad stone* twelve miles away. We found his mind affected to some extent, but had called for his books, saying he wished to straighten them up, for on that day week he was going to die.

He asked Dr. Bailey and myself to be candid and tell him if it was hydrophobia, as he wanted to arrange his business.

On examination of his throat we found tonsils and uvula inflamed, for which we gave him guaiac. We found some difficulty in getting him to swallow water, but he could, with an effort, take it or any medicines offered him.

In the evening his throat seemed better, and the *mad stone* man came and asked that the bite be scarified (which had healed), the *stone* failed to *stick*, and the owner of it announced there was no hydrophobia. Patient's mind seemed brighter, but still could not sleep, and told us he had not had a good night's rest since he was bitten. Temperature, on our first visit, ½° elevation, but became normal and remained so until March 3rd; slept two hours, at which time it raised 1°. We, at this time, had Dr. Novel called in. He diagnosed malaria, but thought it about subdued, thinking elevation of temperature due to irritation, as he was very restless, not being yet able to sleep but little. March 4th, temperature 102°. We decided we had a case of acute mania from the strain on his mind caused from fear of hydrophobia. At this time he seemed hungry, and drank water or stimulants freely; in fact said he was hungry and thirsty. On the night of March 4th he began to sleep very well under celerina, alternately with bromidia, but seemed, when aroused, not to recognize any one. March 5th, still sleeping, and refused food, but would take water or stimulants until 9 p. m.; refused to take water, food or medicine, and we resorted to hypodermics of atropia and morphia to continue rest and quiet him, though he was not violent or hard to keep quiet, and sleep seemed natural. March 6th, still sleeping; takes water and stimulants; pulse fast and weak; temperatnre, 103°, a. m.; 5 p. m., 104°; symptoms generally indicating that death was near, which occurred that night at 11:15, without any convulsions, or threatening of them; all through bowels and kidneys responded to medicine for that purpose.

The above is submitted for the consideration of the profession, and we beg to have your opinion.

